# Charged Ferroelectric Domain Walls for Deterministic ac Signal Control at the Nanoscale

**DOI:** 10.1021/acs.nanolett.1c03182

**Published:** 2021-11-04

**Authors:** Jan Schultheiß, Erik Lysne, Lukas Puntigam, Jakob Schaab, Edith Bourret, Zewu Yan, Stephan Krohns, Dennis Meier

**Affiliations:** † Department of Materials Science and Engineering, Norwegian University of Science and Technology (NTNU), 7034, Trondheim, Norway; ‡ Experimental Physics V, University of Augsburg, 86159, Augsburg, Germany; § Department of Materials, ETH Zurich, 8093, Zurich, Switzerland; ∥ Materials Sciences Division, Lawrence Berkeley National Laboratory, Berkeley, California 94720, United States; ⊥ Department of Physics, ETH Zurich, 8093, Zurich, Switzerland

**Keywords:** Ferroelectric, domain walls, nanoelectronics, improper ferroelectricity, alternating current

## Abstract

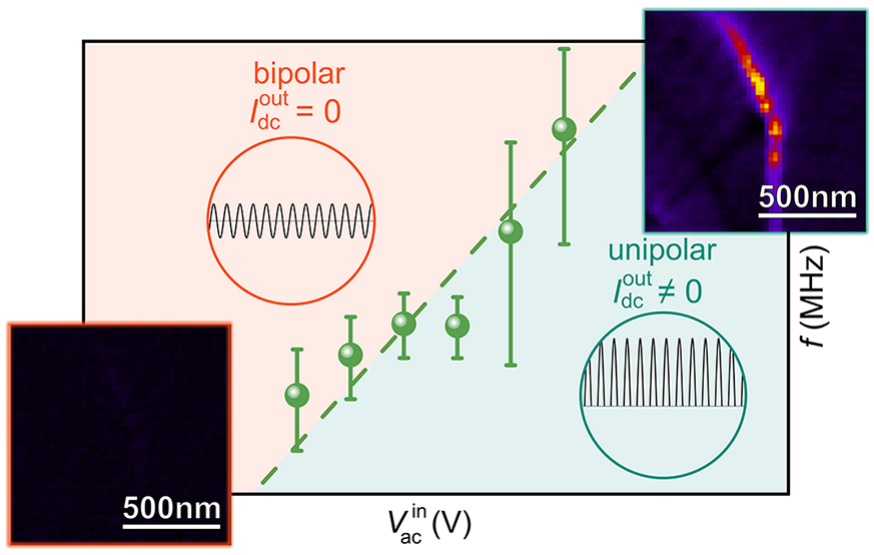

The direct current (dc) conductivity and emergent functionalities at ferroelectric domain walls are closely linked to the local polarization charges. Depending on the charge state, the walls can exhibit unusual dc conduction ranging from insulating to metallic-like, which is leveraged in domain-wall-based memory, multilevel data storage, and synaptic devices. In contrast to the functional dc behaviors at charged walls, their response to alternating currents (ac) remains to be resolved. Here, we reveal ac characteristics at positively and negatively charged walls in ErMnO_3_, distinctly different from the response of the surrounding domains. By combining voltage-dependent spectroscopic measurements on macroscopic and local scales, we demonstrate a pronounced nonlinear response at the electrode-wall junction, which correlates with the domain-wall charge state. The dependence on the ac drive voltage enables reversible switching between uni- and bipolar output signals, providing conceptually new opportunities for the application of charged walls as functional nanoelements in ac circuitry.

## Introduction

Ferroelectric domain walls are excellent candidates for the development of next-generation nanoelectronics, exhibiting a thickness that approaches the unit cell level.^1−3^ Similar to 2D systems such as graphene,^4^ MoS_2_ single layers,^5^ and the LaAlO_3_/SrTiO_3_ heterointerface,^6^ they display unique electronic transport properties^3^ and large carrier mobilities.^7^ In addition to their transport properties, the ferroelectric domain walls are spatially mobile and can be injected and deleted on demand, which enables them to take an active role as reconfigurable elements in, for example, memory,^8,9^ diode^10^ or memristor^11^ devices. Recently, it was demonstrated that intrinsic electronic correlation phenomena at ferroelectric domain walls can be used to control electrical currents, removing the need to write and erase the walls.^12,13^ This observation promoted the idea to develop the walls themselves into devices instead of using them as active elements in much larger electronic components. The approach is intriguing as it breaks the mold of classical device architectures, taking full advantage of the ultrasmall feature size of ferroelectric domain walls. Compared to more than a decade of research on domain-wall devices that operate based on the injection and deletion of domain walls,^14,15^ little is known about the technological potential of stationary walls. Only recently, it was shown that ferroelectric domain walls can be used to emulate the behavior of electronic components at the nanoscale, acting as binary switches^12^ and half-wave rectifiers.^13^ First insight into the electronic properties of domain walls under alternating currents (ac) was obtained for neutral domain walls in the gigahertz regime^16−20^ and applications as tunable microwave devices and acoustic wave filters have been suggested.^21^ In contrast, charged domain walls, which exhibit unusual conduction properties under direct current (dc), have been found to be electronically inactive at high frequencies in the gigahertz regime.^16,22^

In this Letter, we study the electronic response at positively and negatively charged ferroelectric domain walls at intermediate frequencies in the kilo- and megahertz regime. Performing nanoscale spectroscopic measurements on ErMnO_3_, we observe domain-wall specific cutoff frequencies, *f*_c_, at which the current-voltage characteristic of the electrode-wall junction changes from asymmetric to symmetric. By varying the ac voltage amplitude applied to negatively charged walls, we show that the cutoff frequency can readily be tuned by about 1 order of magnitude. This tunability enables reversible switching between uni- and bipolar output signals, facilitating active signal conversion in ac circuits at the nanoscale.

## Results and Discussion

### ac Response of Positively and Negatively Charged Walls

Hexagonal ErMnO_3_ is a ferroelectric narrow band gap semiconductor (p-type, *E*_gap_ ≈ 1.6 eV).^23−25^ The spontaneous polarization is parallel to the *c*-axis (*P* ≈ 6 μC/cm^2^)^26^ and originates from a structural lattice-trimerization,^27,28^ leading to explicitly robust ferroelectric domain walls, including all fundamental types of 180° walls (i.e., neutral side-by-side walls, positively charged head-to-head walls, and negatively charged tail-to-tail walls).^29^ The conduction of the neutral walls has been intensively investigated both in the dc^29−31^ and ac^13,16^ regimes continuously covering frequencies up to the gigahertz range, and their basic electronic properties are well understood. In contrast, at charged domain walls only the dc transport behavior^29,31,32^ and the response at high frequencies in the microwave range^16^ have been studied, whereas their ac properties at intermediate frequencies remain to be explored.

**Figure 1:**
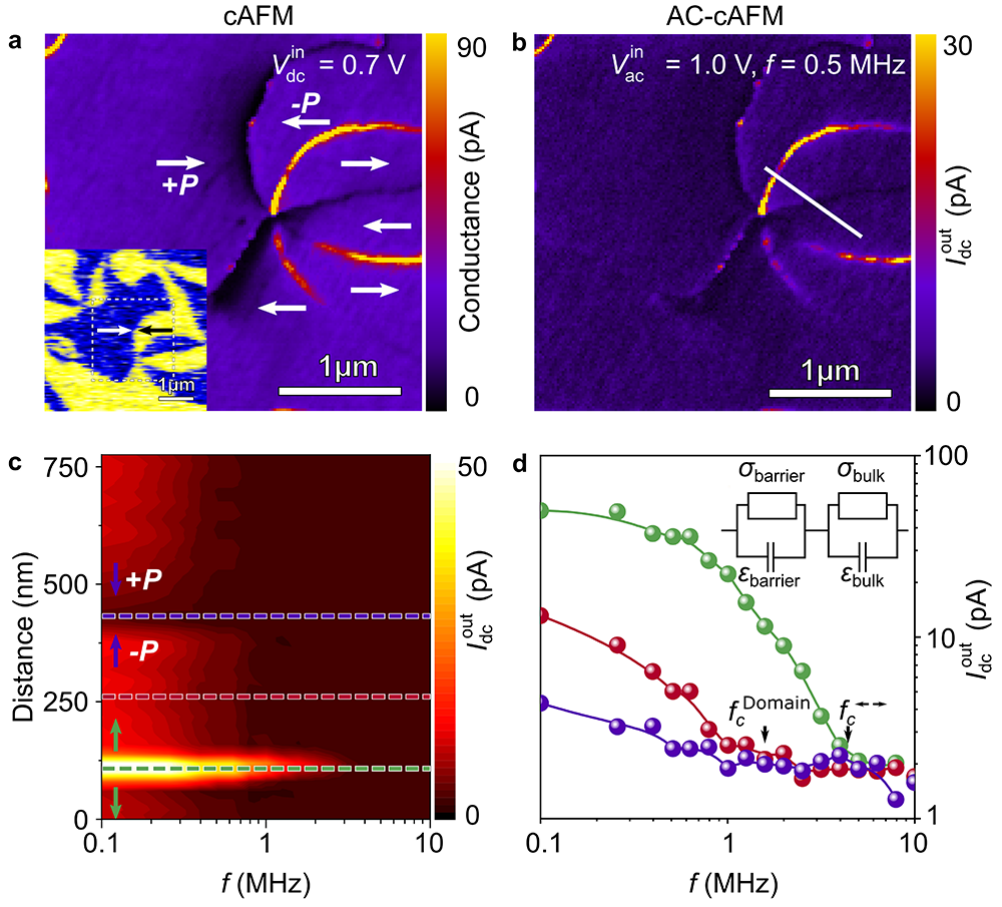
ac response of charged ferroelectric domain walls in ErMnO_3_. (a) cAFM image displaying reduced and enhanced dc conductance at head-to-head and tail-to-tail domain walls, respectively. The polarization direction (indicated by the arrows) is obtained from calibrated PFM data, provided in the inset (blue, +*P*; yellow, −*P*). (b) AC-cAFM scan taken at the same position as the cAFM image in panel a. (c) Frequency-dependent evolution of the AC-cAFM signal along the solid line in panel b. Pronounced AC-cAFM contrast is observed at *f* = 0.1 MHz, vanishing toward increasing frequencies. (d) Local frequency-dependent AC-cAFM response evaluated along the dashed lines in panel c for a domain, a head-to-head, and tail-to-tail domain wall, indicating different cutoff frequencies, *f*_c_, (displayed by arrows) above which the respective signals disappear (*f*_c_^←→^ > *f*_c_^Domain^ > *f*_c_^→←^). The equivalent circuit model in the inset allows for relating the frequency drop to the local intrinsic conductivity,^13^ that is, σ_bulk_^←→^ > σ_bulk_^Domain^ > σ_bulk_^→←^ (barrier conductivity, σ_barrier_; barrier permittivity, ε_barrier_; bulk conductivity, σ_bulk_; and bulk permittivity, ε_bulk_).

The electrical dc transport of a (110)-oriented ErMnO_3_ single crystal (in-plane polarization) is displayed in the conductive atomic force microscopy (cAFM) map in Figure 1a. The orientation of the ferroelectric polarization is indicated by the arrows, determined from the calibrated piezoresponse force microscopy (PFM) image displayed in the inset of Figure 1a. The data shows the established transport behavior,^29^ that is, enhanced conductance (bright) at the tail-to-tail walls and reduced conductance (dark) at the head-to-head walls. In addition, enhanced conduction is observed at nominally neutral domain wall sections, which is consistent with previous work, where the enhancement was attributed to an accumulation of oxygen interstitials^13^ and the sub-surface domain wall orientation.^30^ To investigate the electronic properties of the charged domain walls in the kilo- to megahertz regime, we perform AC-cAFM^13^ scans at the same position. AC-cAFM is a recent spectroscopy method, that allows for probing the dc response (*I*_dc_^out^) under applied bipolar voltages (*V*_ac_^in^) as a function of frequency (Supporting Information and Figure S1).^13^*V*_ac_^in^ describes the amplitude of the bipolar voltage. Figure 1b presents the characteristic AC-cAFM response of both head-to-head and tail-to-tail domain walls at a frequency *f* = 0.5 MHz. In contrast to previous measurements performed under microwave frequencies,^16^ a pronounced response to the ac voltage is detected at the charged domain walls, clearly separating them from the surrounding domains. In addition, the scan in Figure 1b reveals a significant difference in the AC-cAFM response at walls with opposite charge state, showing reduced and enhanced current signals at the head-to-head and tail-to-tail walls, respectively. Thus, the behavior observed in the AC-cAFM scan is consistent with the dc current distribution probed by cAFM (Figure 1a) which is expected to be approached for *f* → 0 Hz.

A systematic analysis of *I*_dc_^out^ at charged domain walls as a function of the frequency of the applied ac voltage is presented in Figure 1c and d. Figure 1c displays *I*_dc_^out^ on a logarithmic frequency scale recorded along the solid line indicated in Figure 1b, featuring a direct comparison of tail-to-tail and head-to-head domain walls with respect to the surrounding domains. At *f* = 0.1 MHz, *I*_dc_^out^ at the insulating head-to-head domain wall is suppressed in comparison to the domains, whereas an enhancement of *I*_dc_^out^ is observed at the tail-to-tail domain wall. With increasing frequency, *I*_dc_^out^ reveals a steplike feature indicating a relaxation process (Figure 1d).^13^ As indicated by the smaller arrows, a cutoff frequency *f*_c_ is defined above which *I*_dc_^out^ reaches a value of less than 1% of the original value. The cutoff frequency *f*_c_ marks a qualitative change in the current-voltage characteristics. Analogous to previous measurements at neutral domain walls in ErMnO_3_,^13^ the ac response at *f* < *f*_c_ is asymmetric due to the Schottky-like tip-sample contact, leading to a nonzero current signal in AC-cAFM.^33,34^ For *f > f*_c_, the AC-cAFM contrast vanishes, indicating symmetric *I*(*V*) characteristics. Furthermore, for the conductive tail-to-tail domain wall the cutoff frequency (*f*_c_^←→^ ∼ 4.0 MHz) is about four times higher than for the domains (*f*_c_^Domain^ ∼ 1.0 MHz). Consistent with its reduced dc conductance (Figure 1a), the cutoff frequency of the insulating head-to-head domain wall is below *f*_c_^Domain^. Because of the much lower current signal than for the domains and the tail-to-tail walls, however, it is difficult to unambiguously quantify *f*_c_^→←^. Thus, we focus on tail-to-tail walls in the later quantitative in-depth analysis.

To rationalize the behavior probed at the charged domain walls, we apply the same equivalent circuit model as used in ref (13), which is illustrated in the inset to Figure 1d. Here, two *RC* elements are connected in series. The domains and domain walls are described by a resistor (with conductivity σ_bulk_) in parallel with a capacitor (with permittivity ε_bulk_). The barrier between tip and sample is described by a barrier conductivity (σ_barrier_) connected in parallel with a capacitor (with permittivity ε_barrier_).^26,35,36^ For *f* < *f*_c_, the transport behavior is dominated by the diode-like tip-sample contact, leading to asymmetric current-voltage characteristics and, hence, a pronounced current signal *I*_dc_^out^ in AC-cAFM. The asymmetric current-voltage characteristics originate from the contact between the probe tip and the p-type semiconducting ErMnO_3_.^23,31^ Because of the different work function of the tip and ErMnO_3_, a Schottky barrier is formed at the tip-sample interface, resulting in rectifying current-voltage behavior as discussed, for example, by Wu et al. in ref (34) for the case of ferroelectric domains in HoMnO_3_. For higher frequencies (*f* > *f*_c_), the *I*_dc_^out^ contrast vanishes, indicating that the tip-sample contact gets short-circuited via the barrier capacitance.^37^ Within our simple equivalent circuit model, the cutoff frequency is defined by the bulk conductivity, σ_bulk_.^13,35,38^ Here, it is important to note that the measured width of the charged domain walls in our local transport measurements is several tens of nanometers due to spreading of the tip-injected currents as discussed in refs (29) and (39). Thus, to evaluate the cutoff frequency for the domain region, *f*_c_^Domain^, we consider a region ∼125 nm away from the walls. The experimentally determined sequence of cutoff frequencies (Figure 1d), *f*_c_^←→^ > *f*_c_^Domain^ > *f*_c_^→←^, thus indicates that σ_bulk_^←→^ > σ_bulk_^Domain^ > σ_bulk_^→←^. This behavior is consistent with dc cAFM measurements (Figure 1a),^29^ where the increase in conductivity at tail-to-tail walls was explained by an enhanced density of mobile holes (majority carriers), which accumulate to screen the negative bound charges at these walls. In contrast, hole depletion occurs to screen the positive bound charges at the head-to-head walls, leading to reduced conductivity relative to the surrounding domains.

To explore the emergence of additional contributions to *f*_c_ beyond the simplistic equivalent circuit model^35,40^ in Figure 1d, we next investigate the effect of varying drive voltages on *f*_c_.

### Voltage-Dependent ac Response at Tail-to-Tail Domain Walls

**Figure 2:**
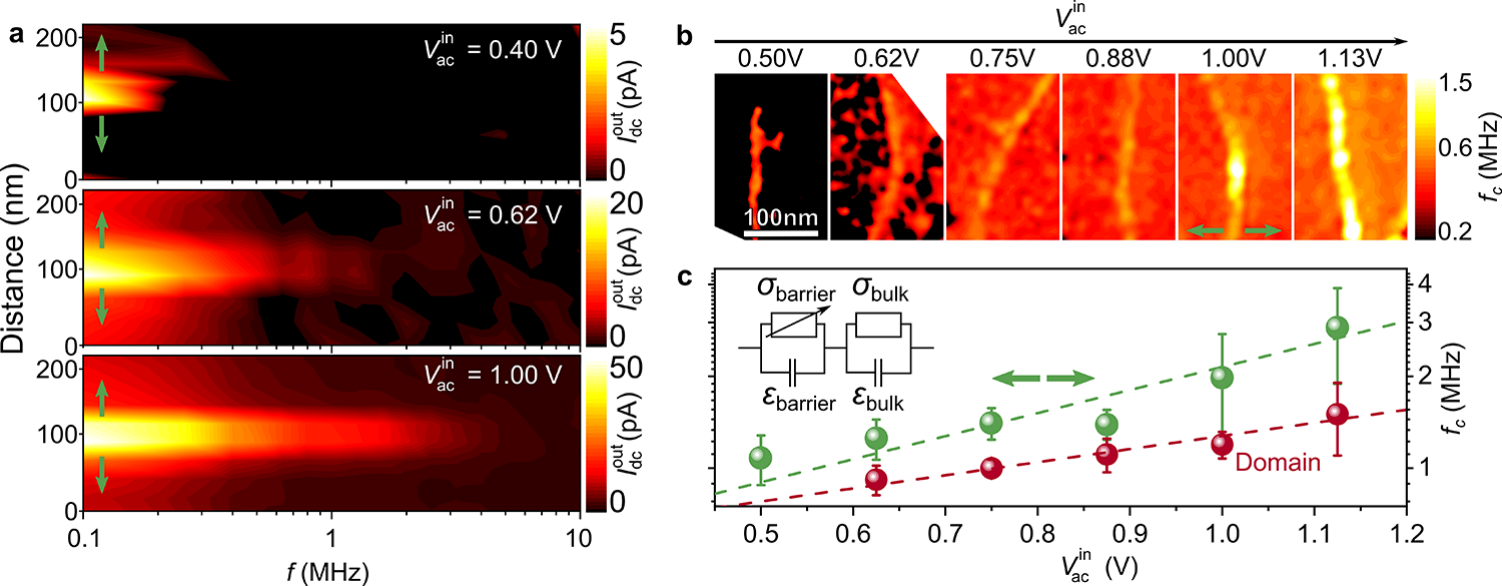
Relation between drive voltage and cutoff frequency. (a) Cross-sectional data showing the frequency dependence of the AC-cAFM response at a negatively charged tail-to-tail domain wall for different voltages. With increasing voltage, *f*_c_ shifts to higher frequencies. (b) Spatially resolved measurements of *f*_c_, recorded at different tail-to-tail domain walls (see also Figure S2). The data is derived from a series of AC-cAFM scans with logarithmically increasing frequencies by fitting the current decay pixel by pixel as explained in the main text and Figure S3. (c) Comparison of the voltage dependence of the cutoff frequencies measured at tail-to-tail walls and in the surrounding domains. Plotted are the mean values; error bars represent the standard deviation. The dashed lines display a guide to the eye. A nonlinear barrier conductivity is introduced into the equivalent circuit model, as displayed in the inset, which allows for capturing the observed voltage-dependent behavior.^42^

The effect of varying drive voltage on the cutoff frequency is presented in Figure 2, showing an overview of frequency- and voltage-dependent AC-cAFM measurements for conducting tail-to-tail domain walls (see Figure S2 for complementary cAFM and PFM data). Figure 2a displays spatially resolved data measured along tail-to-tail domain walls with different *V*_ac_^in^. To avoid possible artifacts caused by repeatedly scanning the same area,^41^ the measurements are performed at different positions on selected walls with comparable dc conductance (see Figure S2 for details). We observe that *f*_c_ increases with increasing *V*_ac_^in^, shifting by more than 1 order of magnitude as *V*_ac_^in^ is raised from 0.40 to 1.00 V. To systematically analyze the correlation between *f*_c_ and *V*_ac_^in^, we record frequency-dependent AC-cAFM maps for a wider voltage range from which we calculate *f*_c_ pixel by pixel as explained in Supporting Information (see Figure S3). Figure 2b displays the resulting cutoff-frequency maps for six tail-to-tail domain walls and the surrounding domains measured at different *V*_ac_^in^. The mean cutoff frequencies obtained for the domains and domain walls are displayed in Figure 2c, and increaseas a function of the applied voltage.

**Figure 3:**
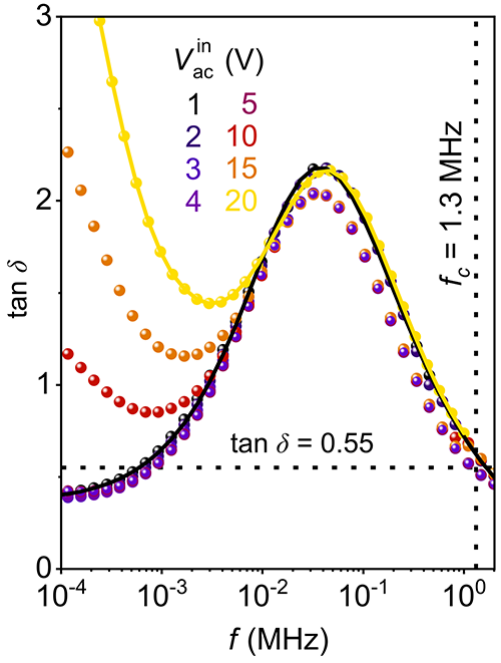
Voltage- and frequency-dependent macroscopic dielectric response. Voltage dependence of the loss factor, tan δ, as a function of frequency, gained on the same sample as used for the local measurements in Figures 1 and 2. The solid lines represent fits of the experimental data (for *V*_ac_^in^ = 1 V and *V*_ac_^in^ = 20 V) utilizing the equivalent circuit model displayed in Figure 1c extended by a frequency-dependent resistance for the bulk as explained previously (see Supporting Information).^26,36^ Analogous to the local measurements (Figures 1 and 2), we define a cutoff frequency, *f*_c_, at which the barrier is short-circuited and the bulk response dominates (tan δ falls below 25% of the maximum value). The identified value of *f*_c_ = 1.3 MHz is in good agreement with the cutoff frequency of the domains found in AC-cAFM (Figure 2c).

To clarify the origin of the additional drive-voltage dependence revealed by AC-cAFM (Figure 2), we perform complementary voltage-dependent macroscopic spectroscopy experiments on the same single crystal. The frequency-dependent loss factor, tan δ, from 10^–4^ to 2 MHz is shown in Figure 3. The voltage and frequency dependence of the dielectric permittivity and the conductivity is displayed in Figure S4. The peak in tan δ at *f* = 5 × 10^–2^ MHz represents the transition regime between the electrode-sample interface and the intrinsic bulk properties of ErMnO_3_.^36,43^ Because of the broadness of the peak,^44^ the electrode-sample interface affects the overall dielectric response even up to much higher frequencies (*f* > 1 MHz, Figure S4). Analogous to the local measurements (Figures 1 and 2), we define a cutoff frequency *f*_c_ (tan δ falls below 25% of the maximum value,^13^Figure 3), which takes the broadness of the peak into account. This value *f*_c_ represents a measure for the frequency at which the contributions from the electrode-sample interface are short-circuited. In the macroscopic measurements, we find a voltage-independent cutoff frequency *f*_c_ = 1.3 MHz, which agrees with the cutoff frequencies identified for the domains in the local AC-cAFM measurements. Note that the shift of *f*_c_ with *V*_ac_^in^ becomes observable in the local AC-cAFM measurements due to a higher local electric field (*E* ≈ 40 kV/cm) compared to the electric fields (*E* ≈ 0.4 kV/cm) used in the macroscopic measurements. As indicated by the solid lines in Figure 3 and Figure S4, the macroscopic dielectric response can be described via fits using the equivalent circuit model displayed in the inset of Figure 1c (see Supporting Information). The analysis shows that σ_barrier_ increases by more than 1 order of magnitude when *V*_ac_^in^ is increased from 1 to 20 V, while all other parameters remain almost unchanged.

This leads us to the conclusion that the voltage-dependent AC-cAFM response in Figure 2 originates from the Schottky-like nature of the tip-sample contact. The latter is corroborated by the equivalent circuit fitting of the macroscopic dielectric data, which indicates a substantial voltage-driven barrier lowering (Figure S4),^45,46^ analogous to previous macroscopic measurements on CaCu_3_Ti_4_O_12_^47^ and BiFeO_3_-based^48^ materials. Thus, the AC-cAFM data gained at the charged domain walls expands previous macroscopic studies on dielectrics to the nanoscale. The voltage dependence of *f*_c_ (Figure 2c) can be captured by introducing a nonlinear voltage dependence of the barrier conductivity into the equivalent circuit model sketched in the inset to Figure 2c, leading to
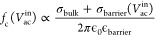
1

In summary, our studies show that the ac characteristics observed at the tail-to-tail domain walls result from their enhanced intrinsic conductivity (Figure 1) in combination with the formation of a voltage-dependent barrier at the electrode-wall junction (Figures 2 and 3).

### Reversible Voltage-Driven Control of the ac Response

**Figure 4:**
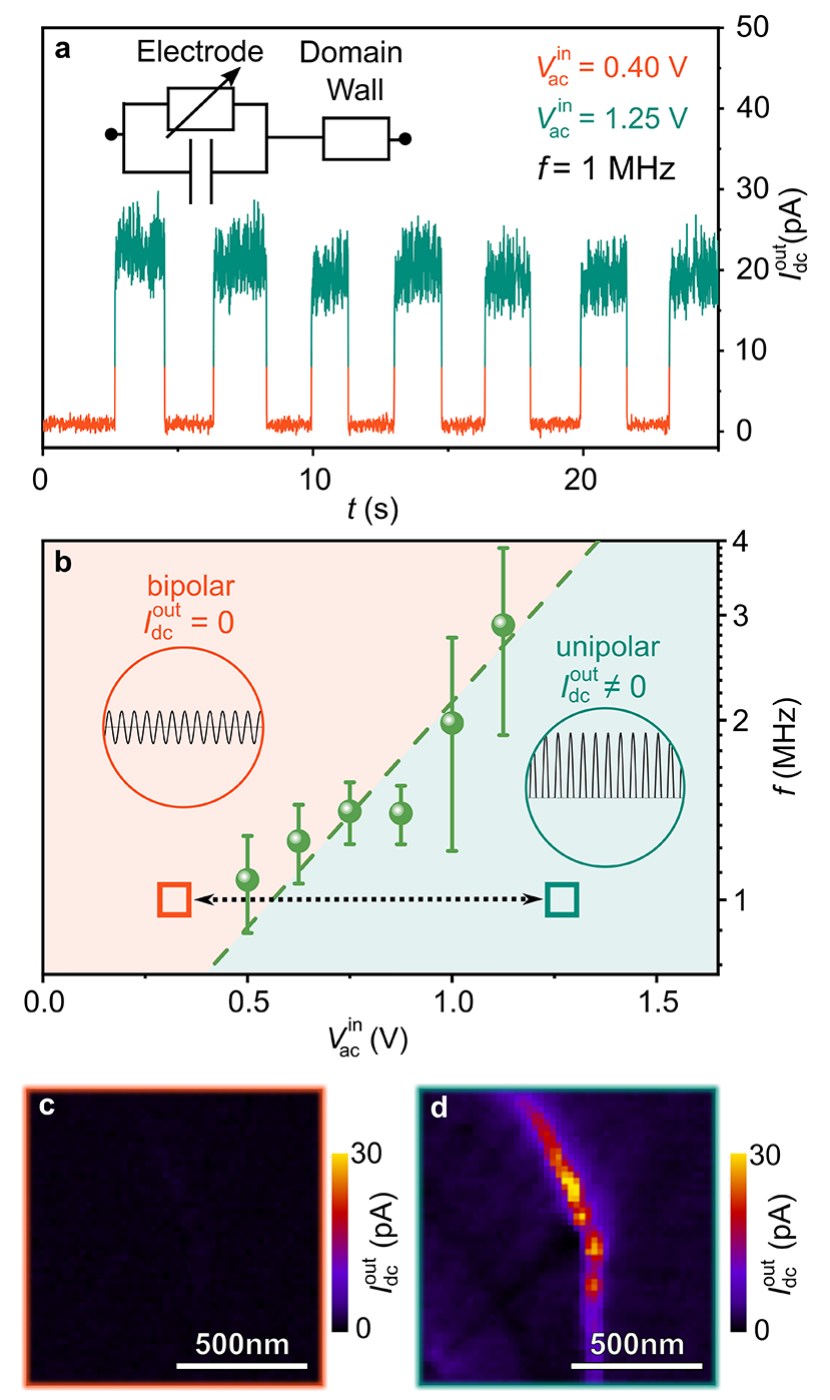
Reversible control of the ac response at tail-to-tail domain walls. (a) AC-cAFM current signal measured with a stationary tip placed on a tail-to-tail domain wall as a function of time over multiple cycles, switching between bipolar (symmetric, *V*_ac_^in^ = 0.40 V, *I*_dc_^out^ = 0) and unipolar (asymmetric, *V*_ac_^in^ = 1.25 V, *I*_dc_^out^ ≠ 0) response at a constant frequency, *f* = 1 MHz. A schematic illustration of a two-terminal ac element emulated by the electrode-wall junction with its equivalent circuit representation^42^ is displayed in the inset. (b) Summary of the electronic response of the ac element in relation to the cutoff frequency and the applied bipolar voltage. The data points and error bars represent *f*_c_ (taken from Figure 2c) and mark the transition between a bipolar (*I*_dc_^out^ = 0) and unipolar (*I*_dc_^out^ ≠ 0) output signal. This transition between the two distinctly different regimes can either be driven by a change in *V*_ac_^in^ (*f* = const.) or, vice versa, by changing *f* (*V*_ac_^in^ = const). The spatially resolved AC-cAFM image gained for the bipolar and unipolar output of the ac element is displayed in c and d, respectively (measured at a constant frequency of *f* = 1 MHz as displayed by the dashed line in panel b).

The relation between *V*_ac_^in^ and the response at the tail-to-tail domain wall allows for controlling the local electronic transport characteristics. In Figure 4, we demonstrate how the junction between the electrode and the ferroelectric domain wall can be utilized to reversibly switch between uni- and bipolar output signals. The AC-cAFM data in Figure 4a is recorded at constant frequency (*f* = 1 MHz) as a function of time, varying the *V*_ac_^in^ repeatedly between 0.40 V (orange) and 1.25 V (green) while keeping the probe tip stationary at the position of the wall. Depending on the applied voltage amplitude, we measure two qualitatively different responses, switching between asymmetric (*I*_dc_^out^ ≠ 0) and symmetric (*I*_dc_^out^ = 0). The two-terminal ac element emulated by the electrode-wall junction and the respective equivalent circuit model is sketched in the inset in Figure 4a. The electrode-wall junction responds symmetrically at low *V*_ac_^in^, whereas an asymmetric response is detected for high *V*_ac_^in^.

The change in voltage allows reversible switching between unipolar and bipolar output. The dependence of *I*_dc_^out^ on both the applied voltage amplitude and frequency is summarized in Figure 4b. The data points in Figure 4b represent the cutoff frequencies obtained from spectroscopic measurements under constant voltages at a tail-to-tail domain wall (Figure 2). The graph emphasizes the existence of two regimes where the electrode-wall junction exhibits qualitatively different electronic responses. The voltage required to transit between these two regimes can be tuned via the frequency of the input signal. Vice versa, facilitated by the voltage-dependent barrier relaxation (Figure S4c and refs (47) and (48)) the cutoff frequency can be selected by adjusting the voltage amplitude of the input signal. Spatially resolved AC-cAFM scans obtained at a tail-to-tail domain wall at *V*_ac_^in^ = 0.4 V and *V*_ac_^in^ = 1.25 V (*f* = 1 MHz) are displayed in Figure 4c and d, respectively, showing the same switching behavior between a unipolar and bipolar response consistent with the data presented in Figure 2a.

## Conclusion

The electronic tunability of the diode-like properties at the electrode-wall junction represents an additional degree of freedom, enabling the design of domain-wall based ac electronic components with ultrasmall feature size. In particular, the involvement of the domain walls ensures that the lateral size is naturally confined with the electronically rectifying area defined by the smallest achievable contact. Application opportunities range from domain-wall based thyrectors that can buffer ripple currents and diodes in transponder circuitry to walls acting as the interconnect between active and passive devices in ac nanoelectronics. In general, the application of charged domain walls in low-frequency nanoelectronics offers several advantages compared to their neutral counterparts.^13^ In contrast to the neutral walls, which owe their transport properties to the accumulation and/or depletion of ionic defects,^13,30^ the conduction at charged domain walls is driven by bound polarization charges, that is, an intrinsic mechanism. The latter implies that defect migration and effects from mixed ionic-electric condictivity^49^ play a less important role compared to neutral domain walls, which is important in order to ensure a reversible and deterministic electronic response at the electrode-wall junction. Furthermore, the bound polarization charges can be used as quasi-dopants^50^ to tune the local conductivity and, thereby, engineering the electronic properties of the electrode-wall junction on demand. Our work introduces charged ferroelectric domain walls as versatile building blocks for ac nanoelectronics in the kilo- to megahertz regime, establishing innovative concepts for domain-wall based nanotechnology and the downscaling of electronic ac components in general.

## Supporting Information Available

Method Section; schematic description of AC-cAFM; positions of the voltage-dependent AC-cAFM scans for Figure 2; determination of the spatial resolution of the cutoff frequency; voltage- and frequency-dependent macroscopic permittivity and conductivity measurements (PDF)

## ABBREVIATIONS

dc: direct current
ac: alternating current
P: polarization
cAFM: conductive atomic force microscopy
PFM: piezoresponse force microscopy.
