# Supplementary material for: Charged Ferroelectric Domain Walls for Deterministic ac Signal Control at the Nanoscale
Source: Nano Lett. 2021 Nov 4;21(22):9560–6. doi: 10.1021/acs.nanolett.1c03182 (PMC8631726; doi:10.1021/acs.nanolett.1c03182)
Supplement: Supplementary file 1 — nl1c03182_si_001.pdf [file nl1c03182_si_001.pdf]

Charged ferroelectric domain walls for deterministic ac signal control at the nanoscale

*Jan Schultheiß[1, *], Erik Lysne[1], Lukas Puntigam[2], Jakob Schaab[3], Edith Bourret[4], Zewu Yan[4,5], Stephan Krohns[2], and Dennis Meier[1, *]*

[1] Department of Materials Science and Engineering, Norwegian University of Science and Technology (NTNU), 7034, Trondheim, Norway

[2] Experimental Physics V, University of Augsburg, 86159, Augsburg, Germany

[3] Department of Materials, ETH Zurich, 8093, Zurich, Switzerland

[4] Materials Sciences Division, Lawrence Berkeley National Laboratory, 94720, Berkeley, CA, USA

[5] Department of Physics, ETH Zurich, 8093, Zurich, Switzerland

*corresponding authors: jan.schultheiss@ntnu.no, dennis.meier@ntnu.no

Supplementary Information

Material. High-quality single crystals were grown using the pressurized floating-zone method[1] and oriented by Laue diffraction with the polarization vector in-plane. The samples with a thickness of about 1 mm were cut and lapped with a 9 µm-grained Al_2O_3 water suspension and polished using silica slurry (Ultra-Sol® 2EX, Eminess Technologies, Scottsdale, AZ, USA) to produce a flat surface with a mean roughness of about 1.55 nm (determined by atomic force microscopy considering a 25x25 µm² scan area).

Local electric characterization. Scanning probe microscopy measurements were performed on an NT-MDT Ntegra Prisma system (NT-MDT, Moscow, Russia). Voltages (a.c. and d.c.) for PFM, cAFM, and AC-cAFM measurements were applied through the bottom electrode using a function generator (Agilent 33220 A, Santa

Clara, CA, USA). All scans were performed using an electrically conductive diamond coated tip (DDESP-10, Bruker, Billerica, MA, USA) with a tip height of 10-15 µm and a maximum tip radius of ≈150 nm. All measurements were carried out at room temperature ($T \approx 25$ °C).

For PFM measurements, the sample was excited using an a.c. voltage ($f = 40$ kHz, $V_{\text{a.c.}}^{\text{in}} = 1.5$ V), while the laser deflection was read out by lock-in amplifiers (SR830, Stanford Research Systems, Sunnyvale, CA, USA). The PFM response was calibrated on a periodically out-of-plane poled $LiNbO_3$ sample (PFM03, NT-MDT, Moscow, Russia). For cAFM scans a d.c. voltage was applied ($V_{\text{d.c.}}^{\text{in}} = 0.7$ V), while the sample response was read out using a low current head (SF005, NT-MDT, Moscow, Russia). In this frame, AC-cAFM is an extension of conventional cAFM measurements.[2] Here an a.c. voltage (0.1 MHz < f <10 MHz, 0.4 V < $V_{\text{a.c.}}^{\text{in}}$ < 1.13 V) is applied to the bottom electrode. The low current head mimics a low pass filter with a cutoff frequency around 1 Hz, making the 0 Hz (also termed d.c. or rectified) component accessible. The recorded analog signal is then transferred to a digital signal and spatial resolution of the 0 Hz component (referred to as $I_{\text{d.c.}}^{\text{out}}$) is provided by the atomic force microscope (a detailed description is provided in Figure S1).

Macroscopic dielectric spectroscopy. Macroscopic dielectric spectroscopy was carried out on the same single crystal also used for local electric characterization. Measurements were performed in a plate capacitor geometry, while both surfaces were coated with silver paint. Measurements were performed using an Alpha Analyzer (Novocontrol, Montabaur, Germany) together with a voltage booster option (HVB300, Novocontrol, Montabaur, Germany), covering a frequency range of 10^{-4} to 10 MHz with varying applied bipolar voltages from $V_{\text{a.c.}}^{\text{in}}$ = 1 – 20 V. The measurement was performed at room temperature (T~25°C). More details can be found in ref. 3. The fits of the macroscopic data were done using an equivalent circuit model, consisting of two RC circuits connected in series to describe the behavior of the bulk and the barrier independent of each other over the entire frequency regime. Note that the conductivity of the second RC element of this circuit (the internal contributions) consists of a resistor representing the intrinsic conductivity (σ_{bulk} in Figure 1d and 2c). In the macroscopic dielectric measurements, an additional contribution to the conductivity for the universal dielectric response (σ_{UDR}),[3, 4] covering the influence of hopping transport on $\sigma'(f) \propto f^n$ with exponent $n < 1$[5, 6] was utilized.

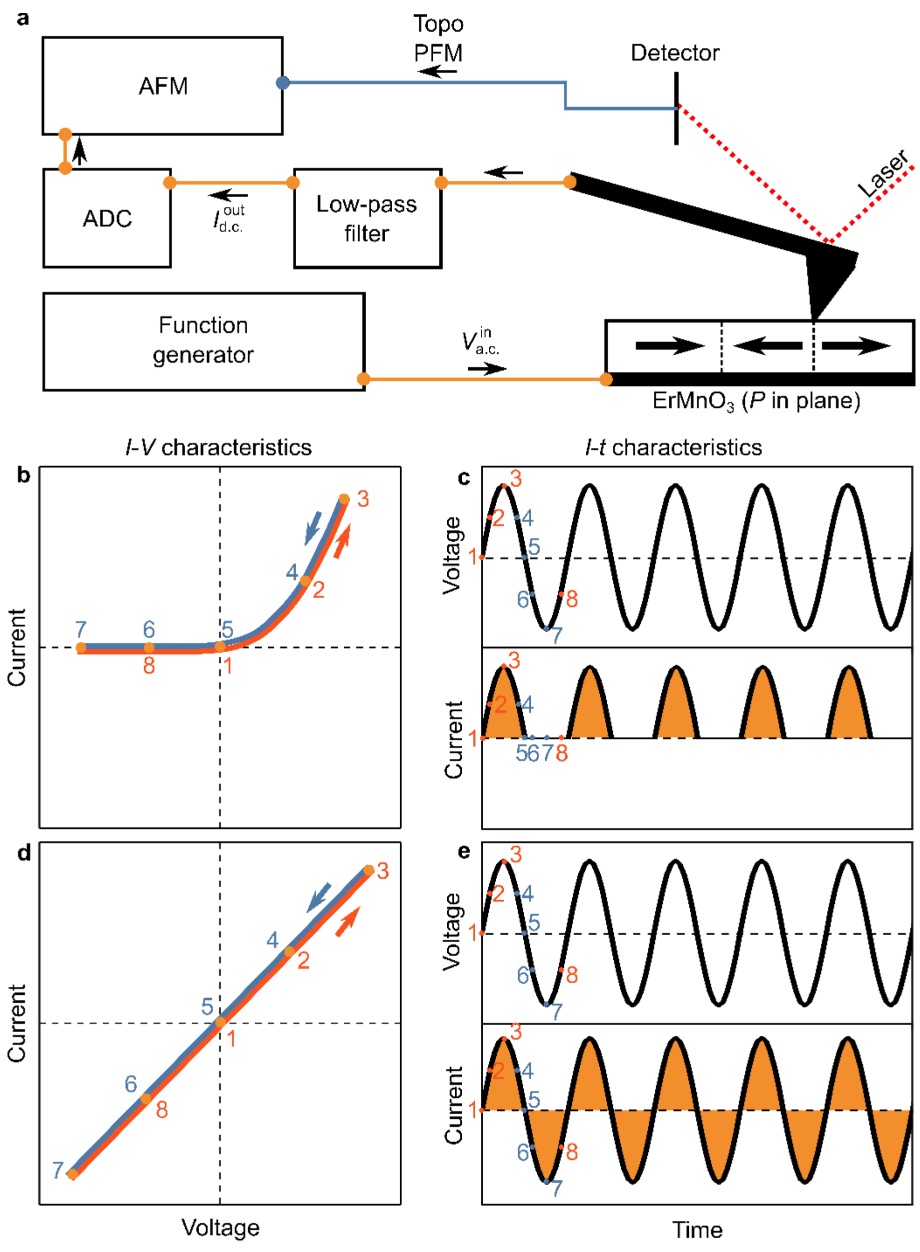

Figure S1. Principle of the AC-cAFM experiment. a) Electronic circuit used in AC-cAFM (wires carrying electrical and mechanical signals are displayed in orange and blue, respectively). An a.c. input voltage (with varying frequency, *f*, and amplitude, $V^{in}_{a.c.}$) is applied to the bottom electrode, while a probe tip is scanned over the surface of the sample in contact mode. Instructive examples for asymmetric and symmetric *I*-*V* characteristics and corresponding AC-cAFM responses are displayed in b), c) and d), e), respectively. b) For a Schottky-like tip-sample contact, the alternating input signal gets rectified.[2, 7] A typical *I*-*V* curve as measured by cAFM on $ErMnO_3$ is schematically depicted in b).[8] c) Illustration showing how the a.c. input voltage ($V^{in}_{a.c.}$) leads to a d.c. current signal due to the asymmetric *I*-*V* characteristics. The AC-cAFM signal ($I^{out}_{d.c.} \neq 0$) represents the time-averaged current response (schematically illustrated by the orange areas). d), e) same as b), c) for the case or a symmetric *I*-*V*

curve, leading to $I_{\rm d.c.}^{\rm out} = 0$. To correlate the voltage- and time-dependent current representations, specific positions are labelled with numbers 1-9 in b)-e).

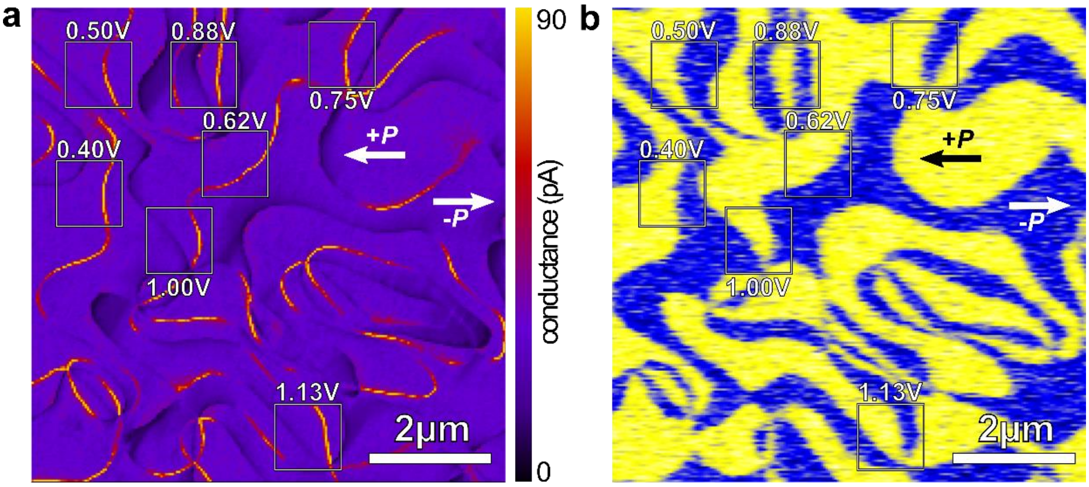

Figure S2. Positions of the voltage-dependent AC-cAFM scans in Figure 2. a) cAFM image ($V_{\rm d.c.}^{\rm in} = 0.7$ V) and b) calibrated PFM overview scan at the same position recorded on a (110)-oriented $ErMnO_3$ single crystal, featuring conductive tail-to-tail and insulating head-to-head domain walls (the polarization orientation, *P*, of the domains is indicated by the arrows). The frequency dependence of the AC-cAFM response of the conductive tail-to-tail domain walls inside the 1.5 x 1.5 μm^2 boxes was systematically investigated under different $V_{\rm a.c.}^{\rm in}$ (the respective $V_{\rm a.c.}^{\rm in}$ is indicated). The results are displayed in Figure 2. The conductive domain walls were chosen for this comparative study since they have a quantitatively comparable d.c. conductance response.

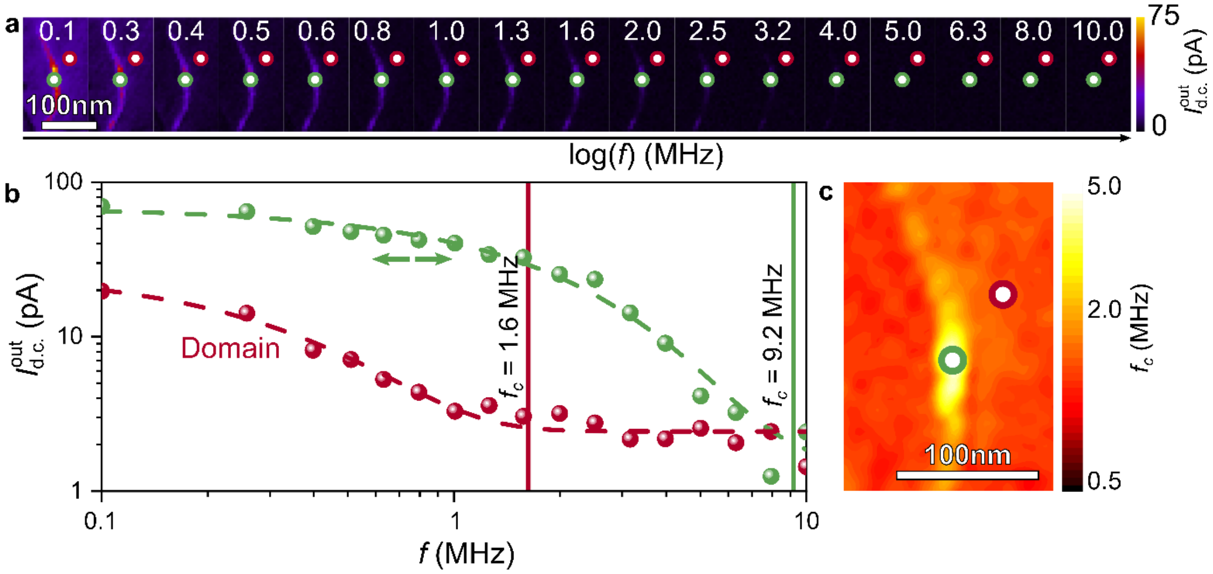

Figure S3. Spatial resolution of the cutoff frequency. a) A series of AC-cAFM scans is displayed at logarithmically increasing a.c. frequencies at the red and green marked positions at the tail-to-tail domain wall and within a domain. The frequency values (in MHz) are displayed in the respective AC-cAFM images. b) The quantitative value of the measured AC-cAFM contrast is evaluated for these two positions (one pixel corresponding to an area, $A{\sim}71 \cdot 10^3\ \mathrm{nm}^2$, estimated by the radius of the tip ($r \approx 150\ \mathrm{nm}$)). An exponential decay function ($f(f) = a \cdot \exp(-f/f_0) + c$) is fitted to the experimental data, with *a*, f_0, and *c* as fitting parameters. Here, f_0 represents the frequency at which 37% of the initial value of the AC-cAFM contrast is reached.[9] Exemplary fits are displayed in b as dashed lines. The cutoff frequency, at which the AC-cAFM contrast vanishes, is finally calculated as $f_c = 5 \cdot f_0$ and is displayed for the two pixels as solid vertical lines in b. The factor 5 was chosen here, because the AC-cAFM signal reaches a value of less than 1 % of its original value.[9] The analysis was automatized for each pixel within the chosen area using a MatLab program to display the cutoff frequency spatially resolved, exemplarily shown for the investigated scan area in c). Figure 2b displays the results for a broad range of voltages.

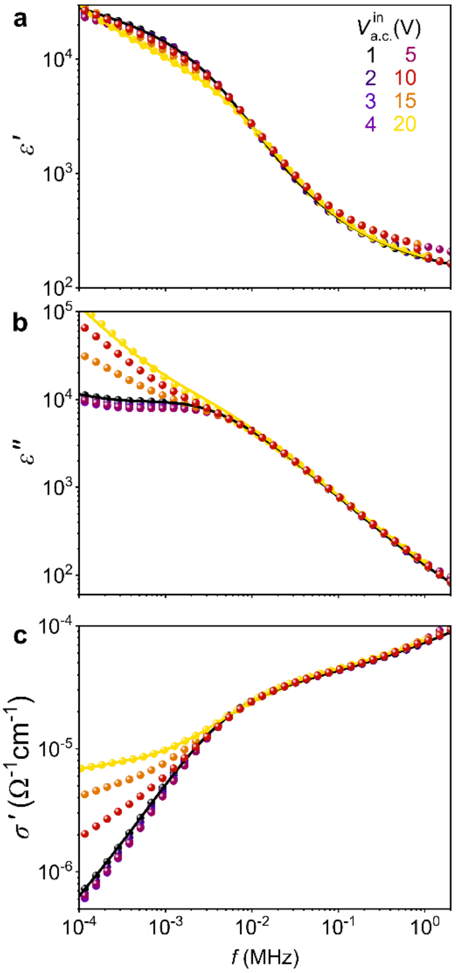

Figure S4. Frequency- and voltage-dependence of the macroscopic permittivity and conductivity. The frequency dependent permittivity (real, ε', and imaginary, ε'', part) and conductivity, σ', are displayed in a), b), and c), respectively. The data is measured under different voltages, $V_{\mathrm{a.c.}}^{\mathrm{in}}$. The solid lines represent fits of the experimental data utilizing the equivalent circuit model displayed in the inset of Figure 2c (extended by the universal dielectric response,[5, 6] as described in the methods section).

REFERENCES

1. Yan, Z.; Meier, D.; Schaab, J.; Ramesh, R.; Samulon, E.; Bourret, E. Growth of high-quality hexagonal $ErMnO_3$ single crystals by the pressurized floating-zone method. *J. Cryst. Growth* **2015,** *409*, 75-79.

2. Schaab, J.; Skjærvø, S. H.; Krohns, S.; Dai, X. Y.; Holtz, M. E.; Cano, A.; Lilienblum, M.; Yan, Z. W.; Bourret, E.; Muller, D. A.; Fiebig, M.; Selbach, S. M.; Meier, D. Electrical half-wave rectification at ferroelectric domain walls. *Nat. Nanotechnol.* **2018,** *13*, 1028-1034.

3. Puntigam, L.; Schultheiß, J.; Strinic, A.; Yan, Z.; Bourret, E.; Altthaler, M.; Kezsmarki, I.; Evans, D. M.; Meier, D.; Krohns, S. Insulating improper ferroelectric domain walls as robust barrier layer capacitors. *J. Appl. Phys.* **2021,** *129*, 074101.

4. Ruff, E.; Krohns, S.; Lilienblum, M.; Meier, D.; Fiebig, M.; Lunkenheimer, P.; Loidl, A. Conductivity Contrast and Tunneling Charge Transport in the Vortexlike Ferroelectric Domain Patterns of Multiferroic Hexagonal $YMnO_3$. *Phys. Rev. Lett.* **2017,** *118*, 036803.

5. Jonscher, A. K. The 'universal' dielectric response. *Nature* **1977,** *267*, 673-679.

6. Lunkenheimer, P.; Krohns, S.; Riegg, S.; Ebbinghaus, S. G.; Reller, A.; Loidl, A. Colossal dielectric constants in transition-metal oxides. *Eur. Phys. J Spec. Top.* **2010,** *180*, 61-89.
7. Wu, W.; Guest, J. R.; Horibe, Y.; Park, S.; Choi, T.; Cheong, S. W.; Bode, M. Polarization-Modulated Rectification at Ferroelectric Surfaces. *Phys. Rev. Lett.* **2010,** *104*, 217601.
8. Meier, D.; Seidel, J.; Cano, A.; Delaney, K.; Kumagai, Y.; Mostovoy, M.; Spaldin, N. A.; Ramesh, R.; Fiebig, M. Anisotropic conductance at improper ferroelectric domain walls. *Nat. Mater.* **2012,** *11*, 284-288.
9. Storey, N., *Electronics: a systems approach*. Pearson Education: 2006, p. 176.
